# Global Trends in mHealth and Medical Education Research: Bibliometrics and Knowledge Graph Analysis

**DOI:** 10.2196/52461

**Published:** 2024-06-04

**Authors:** Yuanhang He, Zhihong Xie, Jiachen Li, Ziang Meng, Dongbo Xue, Chenjun Hao

**Affiliations:** 1 Department of General Surgery, The First Affiliated Hospital of Harbin Medical University, Harbin, China; 2 Key Laboratory of Hepatosplenic Surgery, Ministry of Education, The First Affiliated Hospital of Harbin Medical University, Harbin, China

**Keywords:** mHealth, mobile health, medical education, bibliometric, knowledge map, VOSviewer

## Abstract

**Background:**

Mobile health (mHealth) is an emerging mobile communication and networking technology for health care systems. The integration of mHealth in medical education is growing extremely rapidly, bringing new changes to the field. However, no study has analyzed the publication and research trends occurring in both mHealth and medical education.

**Objective:**

The aim of this study was to summarize the current application and development trends of mHealth in medical education by searching and analyzing published articles related to both mHealth and medical education.

**Methods:**

The literature related to mHealth and medical education published from 2003 to 2023 was searched in the Web of Science core database, and 790 articles were screened according to the search strategy. The HistCite Pro 2.0 tool was used to analyze bibliometric indicators. VOSviewer, Pajek64, and SCImago Graphica software were used to visualize research trends and identify hot spots in the field.

**Results:**

In the past two decades, the number of published papers on mHealth in medical education has gradually increased, from only 3 papers in 2003 to 130 in 2022; this increase became particularly evident in 2007. The global citation score was determined to be 10,600, with an average of 13.42 citations per article. The local citation score was 96. The United States is the country with the most widespread application of mHealth in medical education, and most of the institutions conducting in-depth research in this field are also located in the United States, closely followed by China and the United Kingdom. Based on current trends, global coauthorship and research exchange will likely continue to expand. Among the research journals publishing in this joint field, journals published by JMIR Publications have an absolute advantage. A total of 105 keywords were identified, which were divided into five categories pointing to different research directions.

**Conclusions:**

Under the influence of COVID-19, along with the popularization of smartphones and modern communication technology, the field of combining mHealth and medical education has become a more popular research direction. The concept and application of digital health will be promoted in future developments of medical education.

## Introduction

The rapid development of information and communication technologies in recent years has enabled greater connections to the mobile internet to access any information desired at any time and place, providing favorable conditions for the development of mobile health (mHealth). mHealth offers a full range of health care and medical education services, transcending geographical, time, language, and even organizational barriers [1,2].

mHealth was first defined as “unwired e-med” by Laxminarayan and Istepanian [3] in 2000. In 2003, mHealth was defined as an emerging mobile communication and networking technology for health care systems [4]. mHealth can provide diagnostic and treatment support services through mobile communication devices such as mobile phones, iPads, and personal digital assistants. An mHealth system and its associated app functions have a significant impact on typical health care, clinical data collection, record maintenance, health care information awareness, detection and prevention systems, drug counterfeiting, and theft. Thus, mHealth services have a powerful impact on all health services, including hospitals, care centers, and acute care, and are designed to significantly improve the lives of patients, especially older adults, individuals with physical disabilities, and patients with chronic conditions [4].

Currently, medical resources are extremely unevenly distributed among populations. In many developing countries, medical services have not yet been updated to incorporate current technological capabilities and the level of medical education often lags far behind that of developed countries. The integration and development of mHealth and medical education can help to address this situation. Through mHealth, doctors can provide basic health care and concepts to people living in areas where health services are lacking, and researchers who are experts in the field can share their clinical experience and theoretical knowledge with their peers through mobile communication technologies such as mobile phones. Thus, the widespread adoption of mHealth can not only rapidly raise the level of medical services in a region but can also help to somewhat reduce the gap in health services between different regions of the world and promote the progress of the global health care industry. From the perspective of medical education, medical students have traditionally only been able to acquire theoretical knowledge in the classroom and obtain hands-on experience through clinical practice. With the development and promotion of various medical-related mobile apps, medical education is no longer limited to face-to-face interactions, and more advanced and quality teaching resources can be disseminated through mobile software and other digital means. The combination of mHealth and medical education has provided more access to educational resources for medical students and physician groups at different levels [4].

Bibliometric analysis is a quantitative analysis method combining mathematics and statistics that focuses on the bibliometric characteristics of a research field to help researchers better understand the development trends in the field for guiding more in-depth research [5-7]. As research on mHealth continues to deepen, there have been an increasing number of articles published in the field. However, to date, there has been no bibliometric analysis of research related to the applications of mHealth in medical education. Therefore, in this study, we summarized the literature related to mHealth and medical education to help deepen our understanding of mHealth and identify future directions for its in-depth research in the context of developing medical education.

## Methods

### Ethical Considerations

All of the data collected and analyzed in this study were obtained from online public databases and did not involve any human or animal; thus, ethical approval was not required.

### Data Sources

The Web of Science (WoS) literature database was selected to search, export, and analyze the relevant literature linking mHealth and medical education. Although the concept of mHealth was first proposed in 2000, since it was only officially defined in 2003, we set the start date for the search to 2003 [3,4,8]. We searched the WoS platform on April 2, 2023, selecting the WoS Core Collection, which contains articles included in the SCI (Science Citation Index)-EXPANDED, SSCI (Social Science Citation Index), AHCI (Arts & Humanities Citation Index), CPCI-S (Conference Proceedings Citation Index-Science), CPCI-SSH (Conference Proceedings Citation Index-Social Science & Humanities), BKCI-S (Book Citation Index-Science), BKCI-SSH (Book Citation Index-Social Science & Humanities), ESCI (Emerging Sources Citation Index), CCR (Current Chemical Reactions)-EXPANDED, and IC (Index Chemicus) databases.

### Search Strategy

The search in the WoS Core Collection was performed in advanced search mode and the search option was set to “exact search.” The search terms included a combination of “mHealth,” “mobile health,” and “medical education” as follows: “TS=[(mobile health) OR (mHealth)] AND [medical education].” The time span was from January 1, 2003, to March 31, 2023; the document type was limited to “Articles”; and English was selected as the only language of publication. The first output of the articles retrieved was obtained according to this strategy without setting any other inclusion criteria.

### Data Analysis and Visualization

The literature retrieved based on the search strategy outlined above was exported in both plain-text (txt) and tab-delimited (txt) file formats. Descriptive statistics were obtained using HistCite Pro 2.1 [9]. Microsoft Excel 2021 was used to summarize the results from the HistCite Pro 2.1 analysis quantitatively and present the data graphically. VOSviewer (version 1.6.17) was used for cocitation correlation analysis and knowledge mapping [10]. VOSviewer (version 1.6.17) [11] and Pajek64 (version 5.16) were used jointly to analyze the current state of research and time trends. Visualization of country/region coauthorship trends was achieved using the combined powerful mapping capabilities of VOSviewer (version 1.6.17) and SCImago Graphica (version 1.0.34).

### VOSviewer Software Settings

We used VOSviewer to perform a keyword co-occurrence analysis on the exported documents, setting the unit of analysis to “all keywords” and the counting method to “full counting”; the minimum number of occurrences was set to 10. For the overlay visualization, we utilized Pajek software for classification assistance. In the national and regional coauthorship trends analysis, we set the minimum number of coauthors for each country to 5 in VOSviewer. In the cocited references analysis, we set the minimum number of citations to 10. In the cocited journal sources analysis, we set the minimum cocitation count to 35. In the cocited authors analysis, we set the minimum number of citations to 20.

## Results

### Search Results and Publication Trends

A total of 790 publications related to mHealth and medical education were retrieved based on the search strategy outlined in the Methods, which were analyzed by HistCite Pro 2.1. The local citation score (LCS) and global citation score (GCS) were calculated by the HistCite Pro software based on the information provided in the documents. The LCS refers to the number of times a document is cited within a given topic, reflecting the extent of recognition of research findings within the peer community. The GCS represents the number of times a document is cited across all fields globally, serving as a significant indicator of the interdisciplinary and cross-domain impact of research outcomes. The GCS for the 790 articles was 10,600, with an average of 13.42 citations per article, and the LCS was 96.

**Figure 1.**
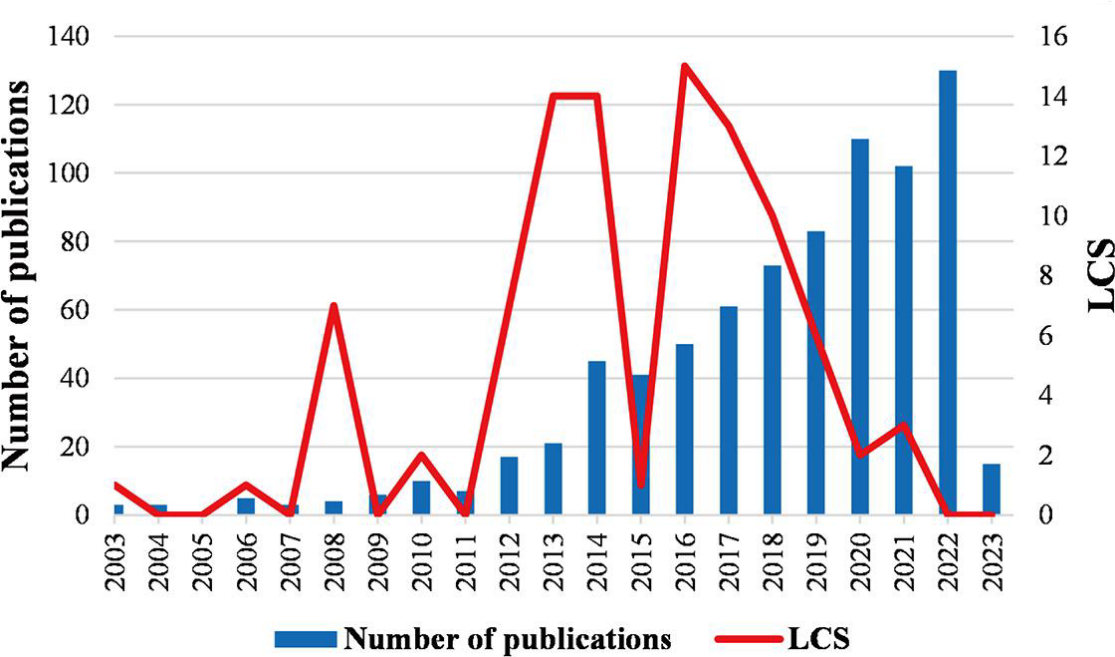
Annual trend in the number of publications and local citation score (LCS) in the field of mobile health and medical education from 2003 to 2023.

Figure 1 shows the number of mHealth and medical education–related publications and the associated changes in the LCS over time. In the last two decades, especially since 2007, the annual number of publications has been steadily increasing year by year. Since 2020, the annual number of publications has exceeded 100, rising to 130 in 2022. However, data for 2023 only include publications from the first 3 months and are thus incomplete, making it difficult to determine the publication trend for that year. In terms of the LCS, the highest value was 15 in 2016, indicating a significant reference value for research in mHealth and medical education in that year. Additionally, there were peaks in the LCS detected in 2008 (7), 2013 (14), 2014 (14), and 2016 (15), indicating that studies in these years had large contributions to the research published in this field in the subsequent years. However, due to limitations of the search time frame, articles submitted in 2022 and 2023 may still be under review and not yet been published (and therefore not yet cited), resulting in an incomplete calculation of LCS values for the past 3 years.

### Contributions of Countries and Institutions

**Figure 2.**
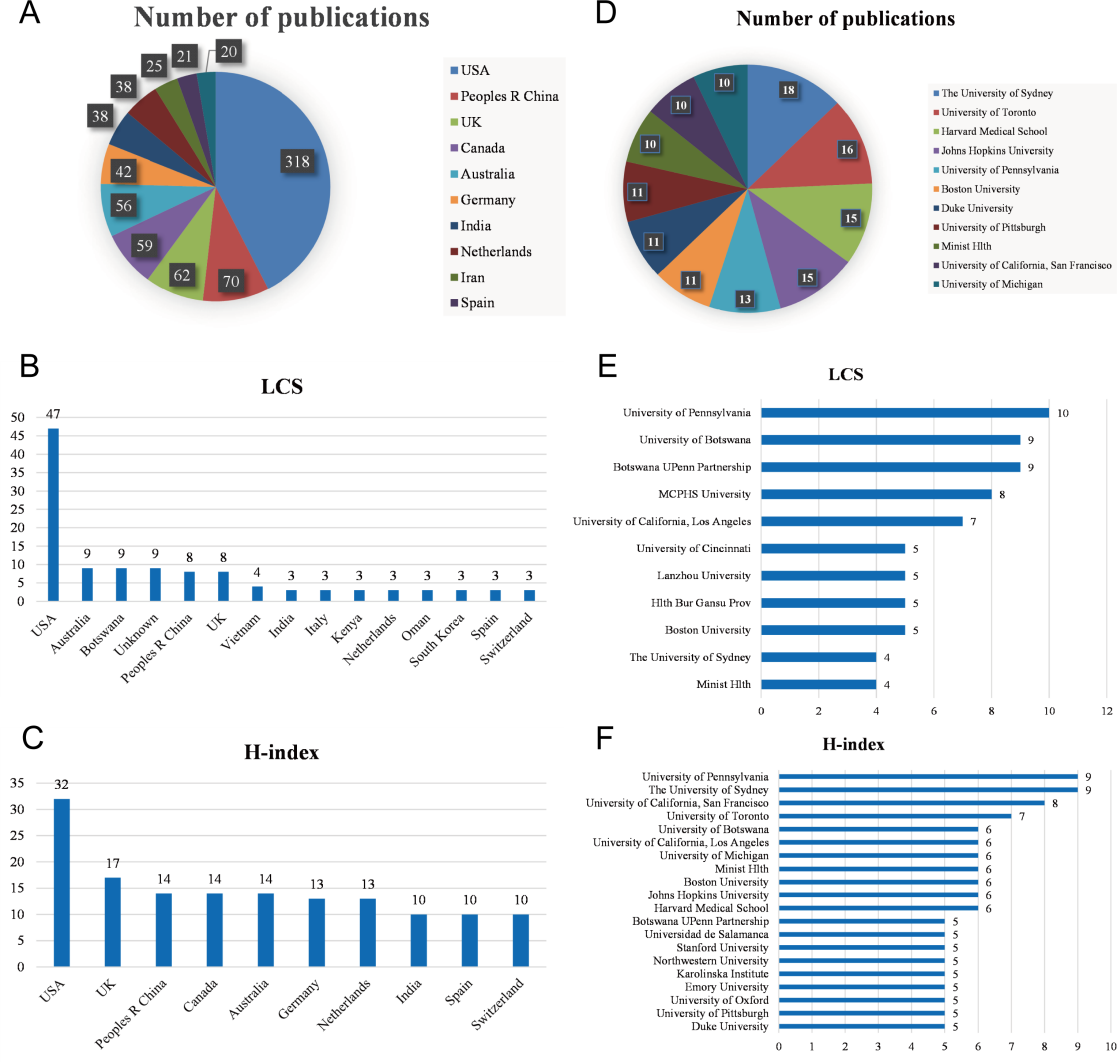
Ranking of top publishing countries and institutions in the field of mobile health and medical education. (A) The top 10 countries with the largest number of publications and their proportions. (B) The top 5 countries with the largest LCS and their proportions. (C) The top 5 countries with the largest H-index values. (D) Institutions with more than 10 publications. (E) The top 6 institutions with the largest LCS. (F) The top 5 institutions with the largest H-index values. Hlth Bur Gansu Prov: Health Bureau of Gansu Province; LCS: local citation score; MCPHS: Massachusetts College of Pharmacy and Health Sciences; Minist Hlth: Ministry of Health; Peoples R China: People’s Republic of China.

We analyzed the top countries and institutions that have published research related to mHealth and medical education. Figure 2A shows the top 10 countries in terms of publication volume, with each country publishing over 20 articles. The United States ranked first with a total of 318 articles, accounting for 40.25% of the total publication volume, representing a contribution far greater than that of other countries. China (n=70) and the United Kingdom (n=62) ranked second and third, respectively. The top 5 countries with respect to the LCS are presented in Figure 2B, with the LCS for the United States reaching 47, which was much higher than that for any other country. Figure 2C lists the top 5 countries in terms of the article H-index, with the United States again ranking first with an H-index of 32; followed by the United Kingdom (17) in second; and China, Canada, and Australia tying for third with an H-index of 14 each. Therefore, the United States leads in both the quantity and quality of publications related to mHealth and medical education, while China and the United Kingdom also rank in the top three for all indicators.

We subsequently analyzed the institutions that published the retrieved articles in this field. Figure 2D shows the institutions with more than 10 publications on the topic, with The University of Sydney ranking first with 18 articles, followed by The University of Toronto (n=16), and Harvard Medical School and Johns Hopkins University tied for third place with 15 relevant publications each. In terms of the LCS, The University of Pennsylvania ranked first with a score of 10 (Figure 2E). Figure 2F compares the top 5 institutions in terms of the H-index, with The University of Pennsylvania and The University of Sydney having the highest H-index of 9 each. Thus, overall, the world’s leading universities such as The University of Pennsylvania and The University of Sydney are producing relatively advanced research in mHealth and medical education, and this institutional-based analysis is largely consistent with the country-based analysis.

### Journal of Publication and Authors

**Figure 3.**
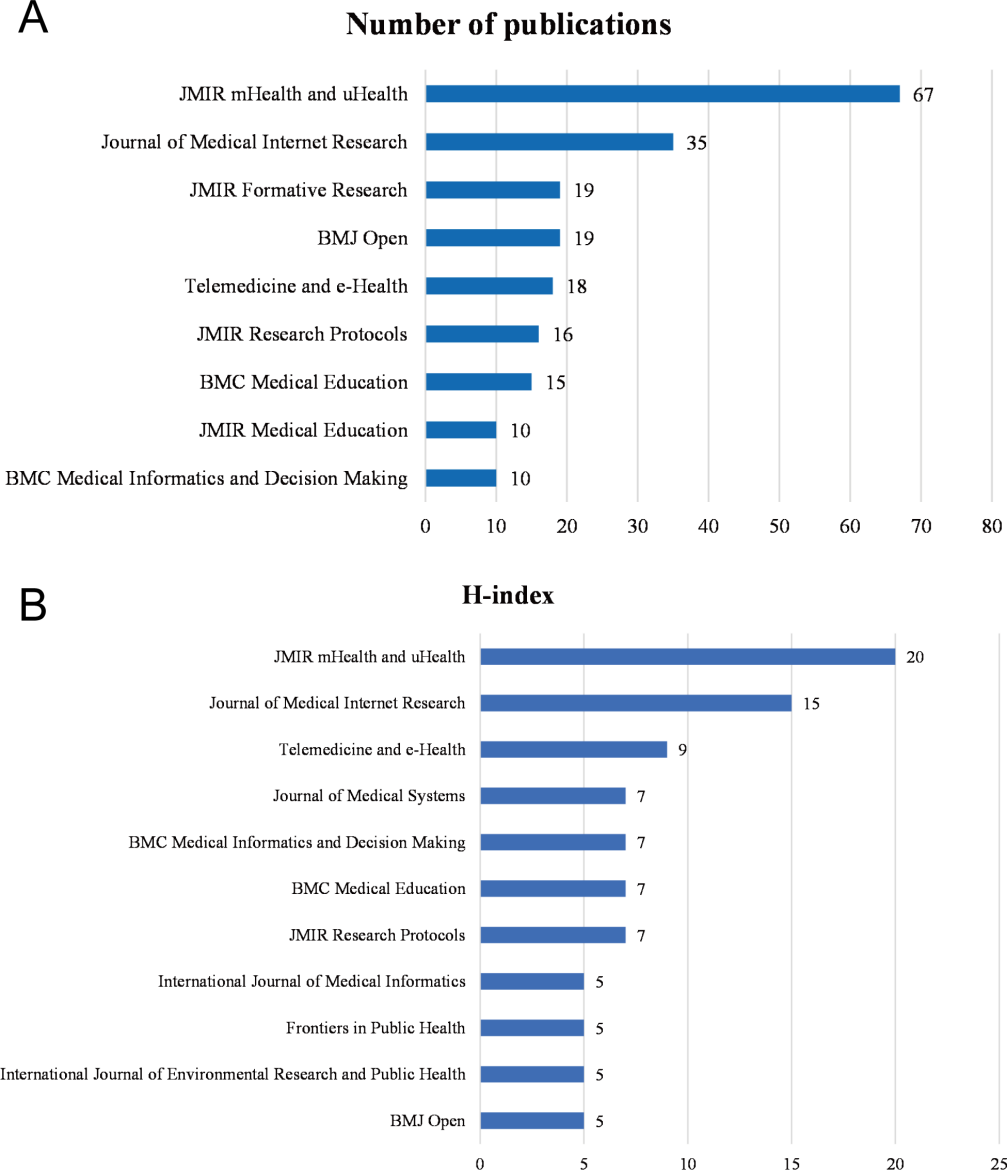
Contributions of journals to the field of mobile health and medical education. (A) Journals with more than 10 publications. (B) Top 5 journals with the largest H-index values.

A total of 420 journals were involved in publishing mHealth and medical education–related articles according to statistics compiled with HistCite Pro 2.1. *JMIR mHealth and uHealth* ranked first with 67 related publications, *Journal of Medical Internet Research* ranked second with 35 articles, and *JMIR Formative Research* and *BMJ Open* ranked third with 19 articles each. Among the journals with more than 10 publications, five are from JMIR Publications (Figure 3A). In terms of the H-index, *JMIR mHealth and uHealth* again ranked first with an H-index of 20, *Journal of Medical Internet Research* ranked second (15), and *Telemedicine and e-Health* ranked third (9) (Figure 3B). This finding demonstrates the comprehensiveness and authority of the JMIR Publications journal series in the field of mHealth and medical education.

The authors with the highest number of publications published 5 articles each, and since most of these authors are repeated coauthors, this field appears to be dominated by a relatively small set of researchers. Table 1 lists the authors with more than 4 articles published along with their LCS and GCS; among them, Littman-Quinn R, Aungst TD, and Kovarik CL are at the top of the list in terms of both the quantity and quality of publications.

**Table 1.** Authors with more than 4 publications and their associated local citation score (LCS) and global citation score (GCS).

Author	Number of publications	LCS	GCS
Deng N	5	0	23
Gill CJ	5	4	33
Halim N	5	4	33
Littman-Quinn R	5	9	163
Schooley B	5	1	60
Aungst TD	4	8	103
Barteit S	4	0	53
Briz-Ponce L	4	0	305
Duan HL	4	0	21
Kim J	4	0	11
Kovarik CL	4	8	136
Li Y	4	0	86
Neuhann F	4	0	53
Scott KM	4	2	101
Williams AL	4	4	33

### Keyword Co-Occurrence and Research Trends

We used VOSviewer to conduct keyword co-occurrence analysis on the exported 790 documents. A total of 3288 keywords were extracted, with 105 keywords meeting the threshold. The 105 keywords were then plotted using VOSviewer for density, network, and overlay visualization.

**Figure 4.**
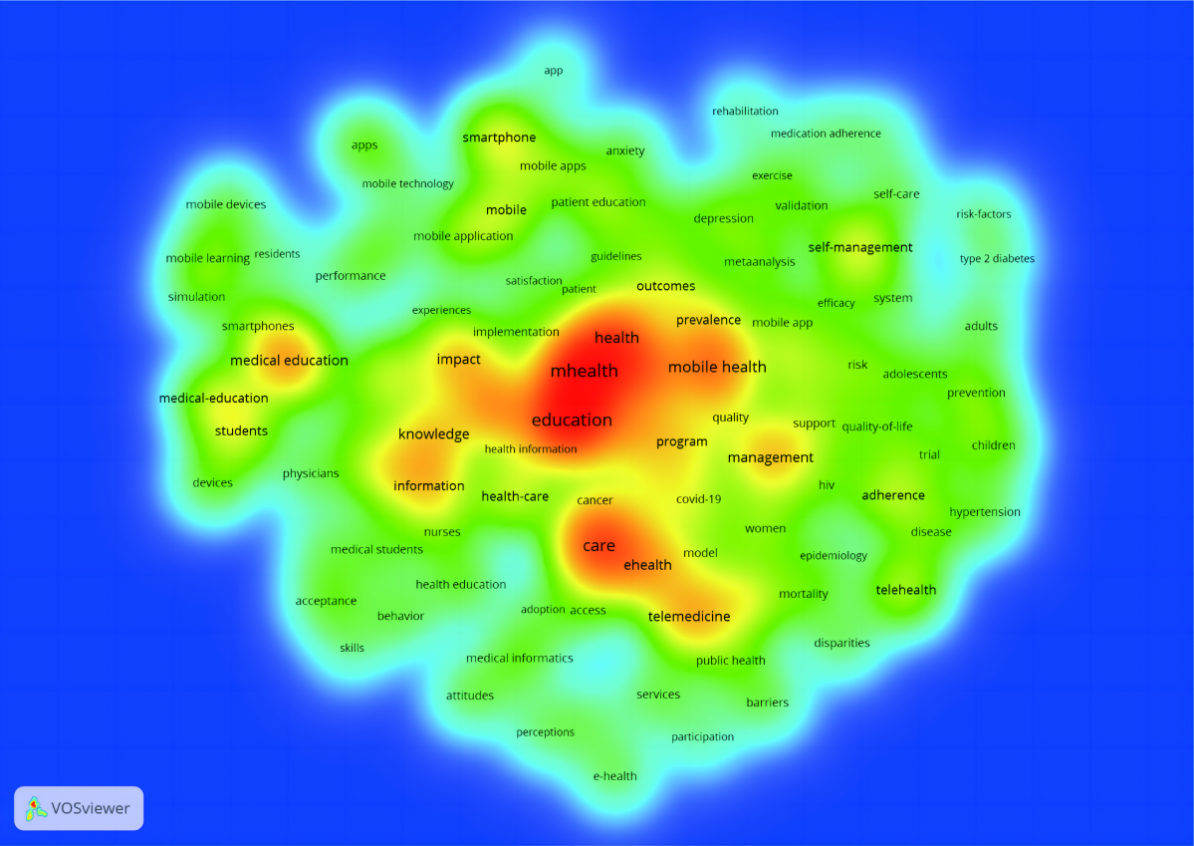
Density visualization of the top 105 keywords. The higher the keyword density, the redder its surrounding color.

Figure 4 shows the density visualization of the 105 keywords, revealing that the majority of research in this field revolves around mHealth or education, which, to a certain extent, verifies the objectivity and scientificity of our search strategy and analysis.

**Figure 5.**
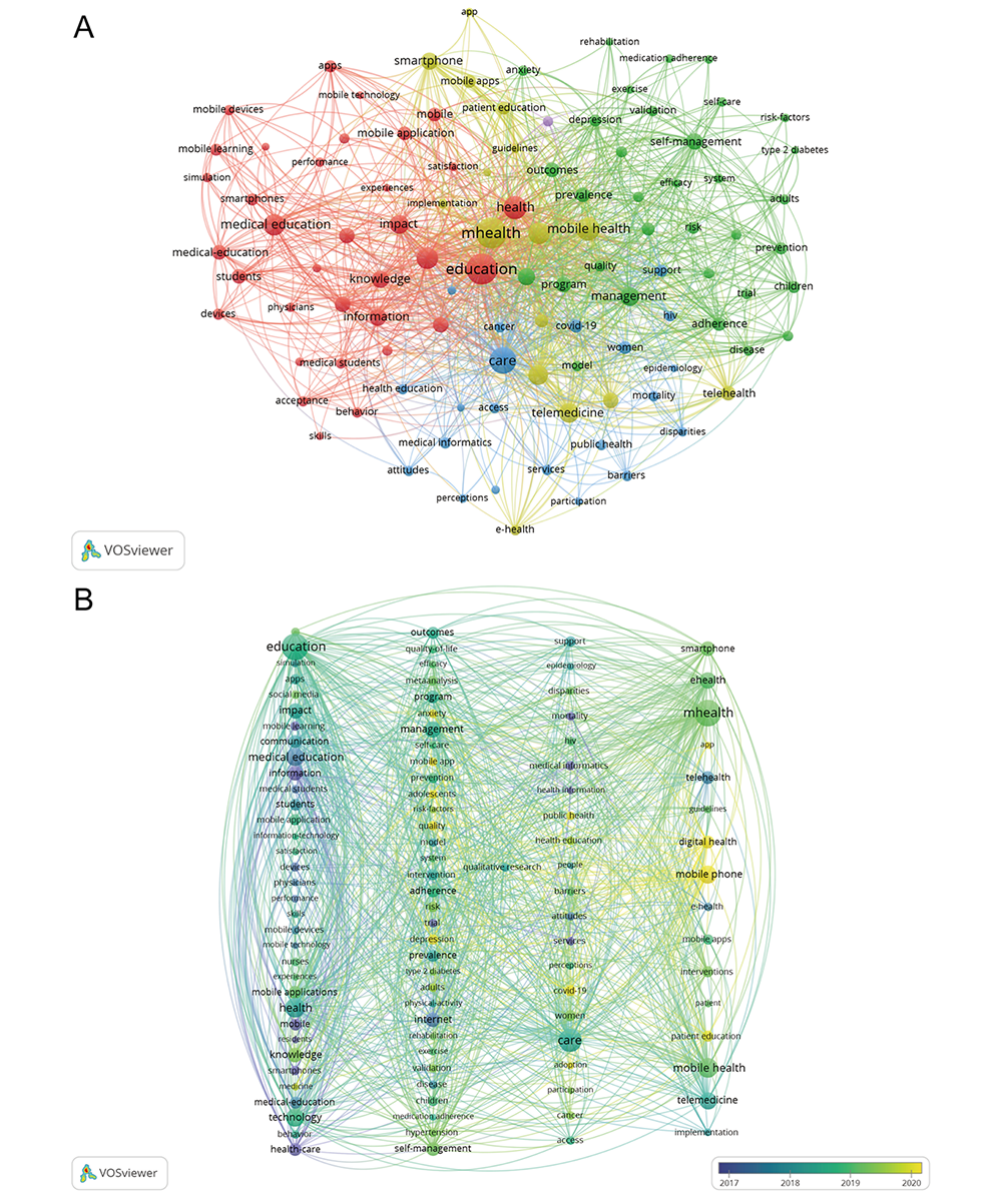
(A) Network and (B) overlay visualization of the 105 keywords.

Figure 5A shows a network visualization of the 105 keywords. A color node can roughly represent a research direction and a larger node area typically indicates a more popular keyword. The software divided all keywords into 5 categories. The red cluster consists of 34 keywords, primarily focusing on clinical medical education (including the keywords “education” and “medical education”), demonstrating that the application of mobile medical software (ie, mobile apps) in medical education and knowledge is widely studied. The green cluster comprises 33 keywords, mainly focusing on the management and development of mobile medical devices and software as well as their application in different age groups through the internet and mobile apps (including the keywords “internet,” “mobile app,” “management,” “outcomes,” “adults,” “children,” and “adolescents”). The blue cluster includes 21 keywords, emphasizing the promotion and education of mHealth in public health and epidemiology (including the keywords “public health,” “medical informatics,” “health education,” “epidemiology,” “HIV,” and “COVID-19”). The yellow cluster contains 16 keywords, primarily investigating the association of mHealth with smartphones, applications in remote diagnosis and treatment, and its role in digital medicine (including the keywords “mHealth,” “smartphone,” “mobile phone,” “telehealth,” “telemedicine,” and “digital health”). As the purple cluster contains only one keyword, “qualitative research,” this serves as a link between various research areas owing to its vague directionality.

Figure 5B presents an overlay visualization of the 109 keywords highlighted in research related to the field of mHealth and medical education. According to the color legend, over time, the main keywords in this research area have gradually shifted from the purple (prior to 2017) to yellow (after 2020) category. This indicates that initially, this field was limited to the understanding and learning of mobile information (including the keywords “information,” “mobile,” and “mobile learning”). With the development and popularity of the internet and mobile devices, their use in medical education began to be promoted (including the keywords “internet,” “mobile devices,” “mobile technology,” and “medical education”). Further, with the development of mobile phones and mobile software, the application of mHealth in medical education is no longer limited to the teaching of professional knowledge to students but is also oriented toward the general public and the promotion of educational medical health concepts among different groups of people (represented by most keywords in the teal-colored small-sized nodes).

In recent years, mHealth has increasingly shifted into the research spotlight with the continuous support of smartphones and a greater inclination toward public health, along with the implementation of inclusive medical services and health communication (yellow small-sized nodes). In the future, increased promotion and use of mHealth care may push digital health (highlighted as “digital health” in yellow in Figure 5, referring to the application of digital technologies such as the Internet of Things, artificial intelligence, and big data in health management) to a focused area of research.

### National and Regional Coauthorship Trends

**Figure 6.**
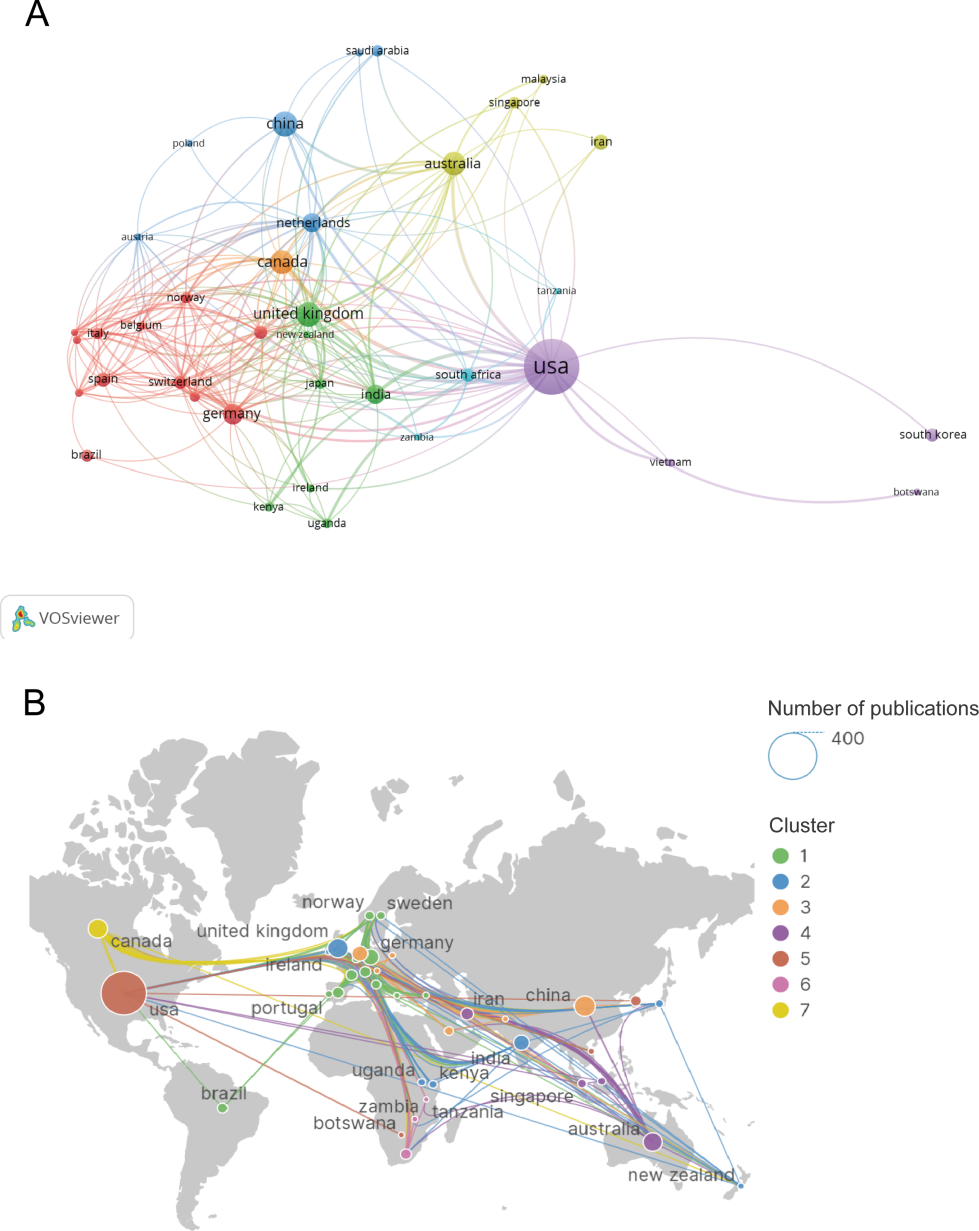
National and regional coauthorship trends. (A) Network map of national coauthorship. (B) Country coauthorship correlations in a world map.

Different countries and regions tend to collaborate on the same research topics rather than working in isolation. VOSviewer identified 37 countries with coauthorship relationships. As shown in Figure 6A, the red clusters (12 countries) have the strongest coauthorship relationships, with the United States (as the country with the highest number of publications) having the most significant coauthorship relationships. We then exported the results of the VOSviewer analysis to SCImago Graphica for further analysis of country coauthorship correlations in a world map (Figure 6B), which provides a clearer visual representation of the strong coauthorship links between countries on all continents, mainly comprising European countries. This map also shows that researchers working in different countries have a large breadth of interactions, even communicating with each other across continents.

### Cocitation Analysis

#### Cocited References

**Figure 7.**
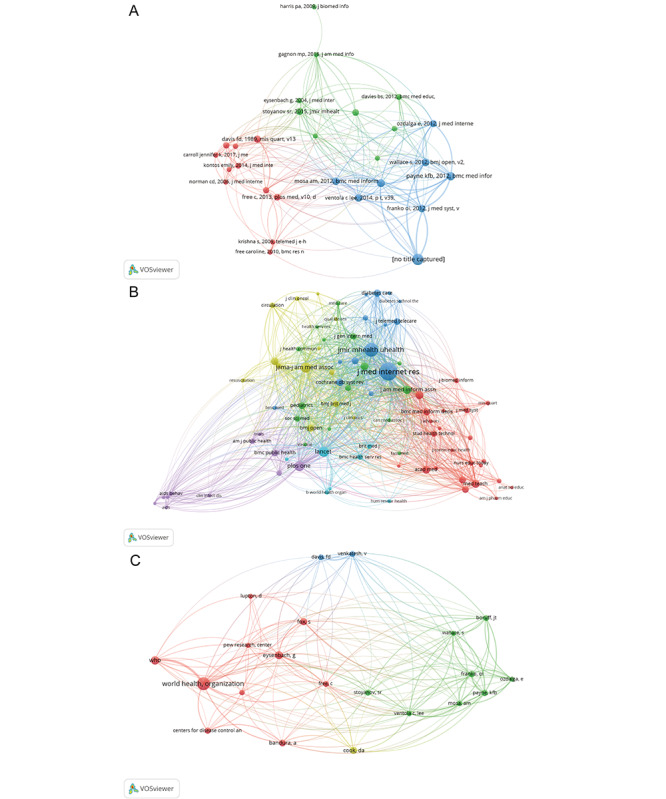
Cocitation analysis. (A) Network map of cocited references. (B) Network map of cocited journal sources. (C) Network map of cocited authors.

Cocited references are an important indicator of the extent to which a particular field is linked to different researchers or research areas. A total of 25,986 cited references were considered valid in VOSviewer, with a total of 28 articles meeting the minimum threshold. These 28 references were divided into three interconnected clusters (Figure 7A), with 11 articles in the red cluster, 9 articles in the green cluster, and 8 articles in the blue cluster. The article “Smartphone and medical related app use among medical students and junior doctors in the United Kingdom (UK): a regional survey” by Payne and colleagues [12], published in *BMC Medical Informatics and Decision Making* [12], showed the highest cocitation frequency, with 28 citations.

#### Cocited Journal Sources

The analysis of cocited journal sources demonstrates the extent to which research in the fields of mHealth and medical education is published in journals that have previously published relevant literature on these topics. A total of 83 journal sources met the minimum threshold (Figure 7B), with the *Journal of Medical Internet Research* having the most cited articles at 898. The 83 journals were divided into six clusters, including 23 journals in the red cluster, 15 in the green cluster, 14 in the blue cluster, 13 in the yellow cluster, 11 in the purple cluster, and 7 in the cyan cluster.

#### Cocited Authors

The number of cocited authors serves as an important indicator for bibliometrics analysis, highlighting the closeness of scholarly relationships and research directions among scholars. In VOSviewer, 22 authors met the minimum threshold of 20,821 citations. According to the network of cocited authors (Figure 7C), a larger area of a color node indicates more citations. The World Health Organization (WHO) had the highest number of citations, reaching 160, reflecting its authority in the field of mHealth combined with medical education. Although each of the four colors represents a different research focus for different authors, the different clusters are not absolutely isolated from each other.

## Discussion

### Principal Results

In this study, we conducted a search of the literature in the WoS Core Collection and obtained 790 relevant articles on mHealth and medical education published from 2003 to 2023 according to the search strategy. In the past two decades, especially since 2007, the number of published papers in this combined field has gradually increased, reaching 130 published papers in 2022. The GCS is 10,600, with an average of 13.42 citations per article, and the LCS is 96. The United States stands out as the country with the greatest application of mHealth in medical education, and most of the institutions with in-depth research in this field are also located in the United States. The depth of research in China and the United Kingdom followed closely behind. Based on current trends, global coauthorship and research exchange will continue to expand. Among the journals publishing research on this topic, JMIR Publications journals have an absolute advantage in this joint field. The 105 keywords identified were divided into five categories pointing to different research directions.

An important indicator of research trends in a field is the number of relevant articles published each year. The results of this analysis show a general upward trend in research in mHealth and medical education, a field that has received a great deal of attention in recent years. As of 2020, research in this joint field can be divided into two phases: the nascent phase and the stable growth phase. The nascent phase spans from the introduction of mHealth in 2003 to 2007 when the model of combining mHealth and medical education was first proposed and associated research was in its infancy, as represented by the small number of relevant articles published in this period. The period from 2007 to 2020 represents a phase of steady growth, with a gradual increase in the number of relevant research articles. In terms of the LCS, there were four peaks detected in 2008, 2013, 2014, and 2016, respectively. Considering the annual publication volume over the entire period of mHealth research, it can be inferred that the research achievements in 2008 played a crucial role in the development of mHealth applications in medical education.

From Figure 5B, it can be seen that the main keywords representing the direction of mHealth before 2017 were “health care,” “internet,” and “information”; however, after 2019, the main keywords changed to “mobile phone,” “mHealth,” and “education,” indicating that the direction of mHealth development has been changing in recent years. This may be due to the popularity of smartphones, development of mobile software, spread of the internet, and rapid development of communication technology. mHealth has evolved from an initial focus on understanding and learning about mobile information and health care information to a combination of mHealth and mobile devices for research and medical education. On January 9, 2007, Steve Jobs, as the Chief Operating Officer of Apple, presented the iPhone 2G and its operating system iOS to the world. This event triggered the rapid development of smartphones and associated apps, as well as the emergence of new mobile platforms. Likely due to these breakthroughs in smartphones and mobile-related technologies, mHealth began to enter the minds of researchers, attracting the attention of scientists worldwide, and thus the number of annual publications related to mHealth began to rise steadily. In addition, the rapid development of communication technology, increasing popularity of smartphones, and development of mobile software provided a suitable platform for medical schools, hospitals, and research institutions in different regions to collaborate and communicate with each other.

On March 11, 2020, the WHO announced COVID-19 as a global pandemic caused by SARS-CoV-2, which affected the daily lives of billions of people [13-15]. The COVID-19 pandemic not only posed a serious challenge to global medical care systems [16] but also limited access to learning and education, with most students having to access knowledge via the internet using communication devices such as mobile phones, iPads, and computers at home. This led to the rapid development of online teaching and learning software, and ultimately accelerated the integration of mHealth and medical education. Consequently, the number of mHealth-related research articles exceeded 100 in 2020 and rose to 130 in 2022. The development and application of 5G mobile technology and the rapid development of online teaching–related software collectively contributed to the deeper integration of mHealth and medical education [17]. Analysis of keyword clusters (Figures 4 and 5A) showed that mHealth research in the last two decades can be roughly divided into four clusters: a clinical education–related cluster, an mHealth equipment and software–related cluster, a health care and public health mission cluster, and a telemedicine cluster. The development of the discipline requires mutual cooperation with other fields. Promoting the integration and development of mHealth and medical education is extremely important to improve the health care conditions in less developed areas such as developing countries and to promote the common development of the world’s health care standards, which is in line with the WHO’s aim to improve the health of people around the world as much as possible.

The high number of citations in this joint field is somewhat indicative of the quality of the research cited. The study by Payne et al [12] received a particularly high number of citations, indicating its significant impact on medical education and mHealth. This study found that medical students and physician groups enjoy acquiring theoretical knowledge through an mHealth teaching model, which is consistent with the overall findings of this bibliometric analysis. In terms of researchers, the WHO has the highest number of cited articles in the field of mHealth combined with medical education, which not only reflects the authority of the organization but also shows the importance the WHO attaches to mHealth combined with medical education. The top three cited journals for mHealth and medical education research are *JMIR mHealth and uHealth* (impact factor 4.948, Q1), *Journal of Medical Internet Research* (impact factor 7.077, Q1), and *BMJ Open* (impact factor 3.007, Q2). According to their impact factors obtained from Journal Citation Reports 2022 [18], these three journals are considered Q1 and Q2 journals, indicating their strong contributions to their respective fields. The cocitation analysis demonstrated the authority of *Journal of Medical Internet Research* in the field of mHealth and medical education research, with an annual volume of 318 articles, an LCS of 47, and an H-index of 32. Although the United States is clearly the world leader in mHealth and medical education, making a significant contribution to the field, academic exchanges between different countries are also ongoing.

### Limitations

There are limitations of our study that should be acknowledged. First, data completeness may be inadequate; although the WoS database has the most complete coverage of articles, our literature search was limited to the English language, which may have resulted in the omission of some key information for some countries where research was published in other languages. In addition, the search strategy was limited to the string “TS=[(mobile health) OR (mHealth)] AND [medical education]” and therefore may not have been sufficiently comprehensive. Second, we only searched under the category “Articles,” which may have also led to missing relevant publications in other formats.

### Conclusion

Bibliometric analysis indicates that mHealth-related research has been growing at an accelerating rate over the last two decades. In the area of combining mHealth and medical education, the WHO is playing an important leadership role, with many researchers following suit. With the influence of COVID-19, the spread of smartphones, and constant developments in modern communication technologies, the field of combining mHealth and medical education is becoming increasingly popular, and the concept and application of digital health will be promoted in the future drive for medical education.

## Abbreviations

AHCI: Arts & Humanities Citation Index
BKCI-S: Book Citation Index-Science
BKCI-SSH: Book Citation Index-Social Science & Humanities
CCR: Current Chemical Reactions
CPCI-S: Conference Proceedings Citation Index-Science
CPCI-SSH: Conference Proceedings Citation Index-Social Science & Humanities
ESCI: Emerging Sources Citation Index
GCS: global citation score
IC: Index Chemicus
LCS: local citation score
mHealth: mobile health
SCI: Science Citation Index
WHO: World Health Organization
WoS: Web of Science
